# Supplementary material for: Premotor cortex uses a compositional neural geometry to plan words
Source: bioRxiv. 2026 Apr 30:2026.04.27.721195. Preprint. [Version 1] doi: 10.64898/2026.04.27.721195 (PMC13142457; doi:10.64898/2026.04.27.721195)
Supplement: 1 [file NIHPP2026.04.27.721195v1-supplement-1.pdf]

bioRxiv preprint doi: https://doi.org/10.64898/2026.04.27.721195; this version posted April 30, 2026. The copyright holder for this preprint (which was not certified by peer review) is the author/funder, who has granted bioRxiv a license to display the preprint in perpetuity. It is made available under aCC-BY 4.0 International license.

**Session ID**	**t12.2022.09.27**	**t15.2024.06.30**	**t15.2024.09.13**	**t12.2025.05.06**	**t12.2022.10.25**
**Cue type**	Nonsense word	Nonsense word	Nonsense word	Real word	Nonsense word
**Sequence Types**	$C_1VC_2VC_3$<br>C_1VC_2<br>C_1V<br>VC_1	$C_1VC_2VC_3$	$C_1VC_2VC_3$<br>C_1VC_2	W_1 W_2 W_3	$C_1VC_2VC_3VC_4V$<br>C_1VC_2V C_3VC_4V
**Sequence elements**	C: K, N, SH, T<br>V: AH	C: K, N, SH<br>V: AH	C: K, N, SH<br>V: AH	clouds, vanish, abruptly	C: K, N, SH<br>V: AH
**Total conditions**	89	28	37	28	163
**Trials per condition**	18	21	15	18	7
**Delay duration (s)**	2.5	1.5 - 3.5	2-4.5	5	2.5
**Go duration (s)**	2 - 2.25	Self-paced (2 - 15)	Self-paced (2 - 45)	4	2.5
**Figure**	Fig 1, 2, 3, 4, S2, S3, S4, S5, S6, S7, S8	Fig 1, 2, 4, S2, S7, S8	Fig 3, S6,	Fig 5	Fig 5

Table S1: Summary of all data collection sessions. Differences between sessions reflect differences in participant abilities and comfort. For example, T15 requested go-periods be self-paced to allow time to regulate breathing between trials. Sequence type notation indicates sequence elements as consonant (C), vowel (V), or word (W); spaces indicate an auditory pause between words; subscripts indicate sequence elements that vary across conditions. All cue designs were full factorial and only t12.2022.10.25 did not include a do-nothing condition. Delay duration includes both audio cue presentation and a post-cue preparatory period.

bioRxiv preprint doi: https://doi.org/10.64898/2026.04.27.721195; this version posted April 30, 2026. The copyright holder for this preprint (which was not certified by peer review) is the author/funder, who has granted bioRxiv a license to display the preprint in perpetuity. It is made available under aCC-BY 4.0 International license.

Supplement

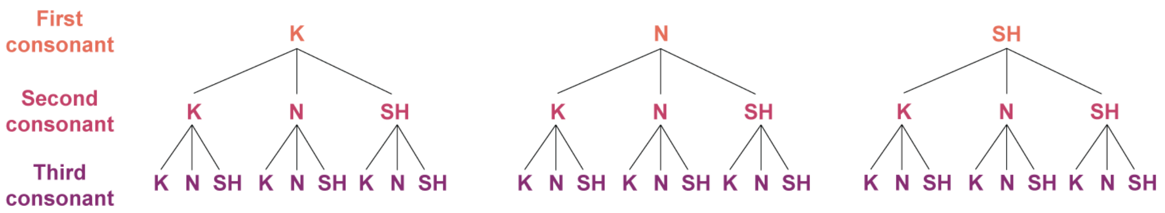

Figure S1: Schematic of full factorial cue design from a subset of consonants. Construction of a full-factorial cue set for 3 consonants (K, N, SH) in 3 positions creates a total of 27 total conditions. Related to Figure 1.

bioRxiv preprint doi: https://doi.org/10.64898/2026.04.27.721195; this version posted April 30, 2026. The copyright holder for this preprint (which was not certified by peer review) is the author/funder, who has granted bioRxiv a license to display the preprint in perpetuity. It is made available under aCC-BY 4.0 International license.

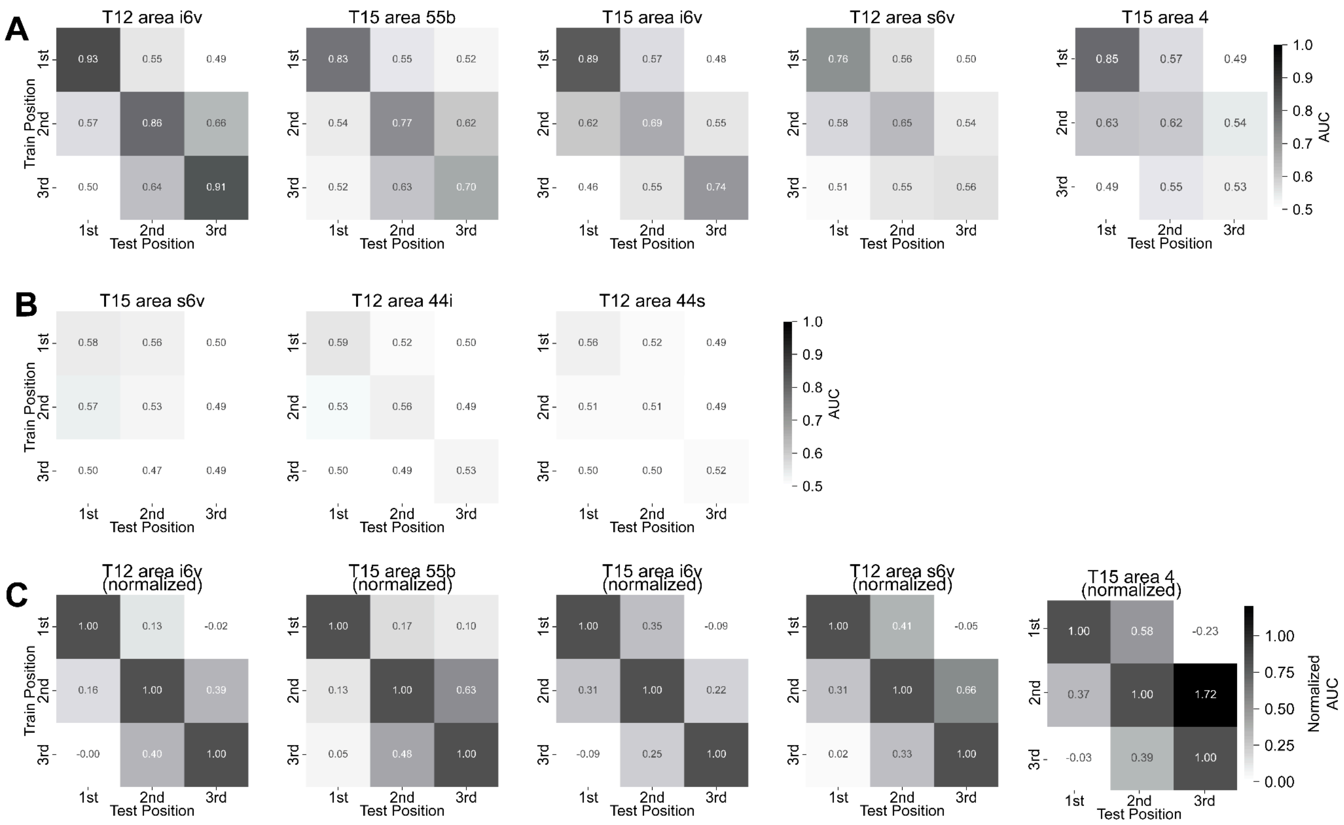

Figure S2: Cross-position generalization of phoneme decoding across all recorded arrays (related to Figure 2). A) For each microelectrode array, multiclass LDA decoders were trained to predict the consonant identity at each sequence position from preparatory neural activity and tested on held-out data labeled by all other positions, quantifying the degree to which phoneme representations generalize across positions (same analysis as Figure 2A; T12 area i6v reproduced here for comparison). **B)** Same analysis as panel **A** for arrays with weaker or absent preparatory tuning to phoneme sequences. **C)** Because within-position decoding performance can vary across positions, raw cross-position AUC values can obscure how well representations generalize relative to the performance ceiling of each position (e.g., in T15 area 4, 1st-position AUC is substantially higher than 2nd-position AUC). To account for this, cross-position AUC values were normalized by within-position performance (column-wise), using the ratio of above-chance cross-position to above-chance within-position AUC: (AUC_{cross} − 0.5) / (AUC_{within} − 0.5), such that a value of 1 indicates generalization equal to within-position performance and 0 indicates chance-level generalization. After normalization, generalization between the 2nd and 3rd positions is consistently stronger than between the 1st and 2nd positions in both T12 area i6v and T15 area 55b, consistent with the pattern of cosine similarities reported in Figure 2F.

bioRxiv preprint doi: https://doi.org/10.64898/2026.04.27.721195; this version posted April 30, 2026. The copyright holder for this preprint (which was not certified by peer review) is the author/funder, who has granted bioRxiv a license to display the preprint in perpetuity. It is made available under aCC-BY 4.0 International license.

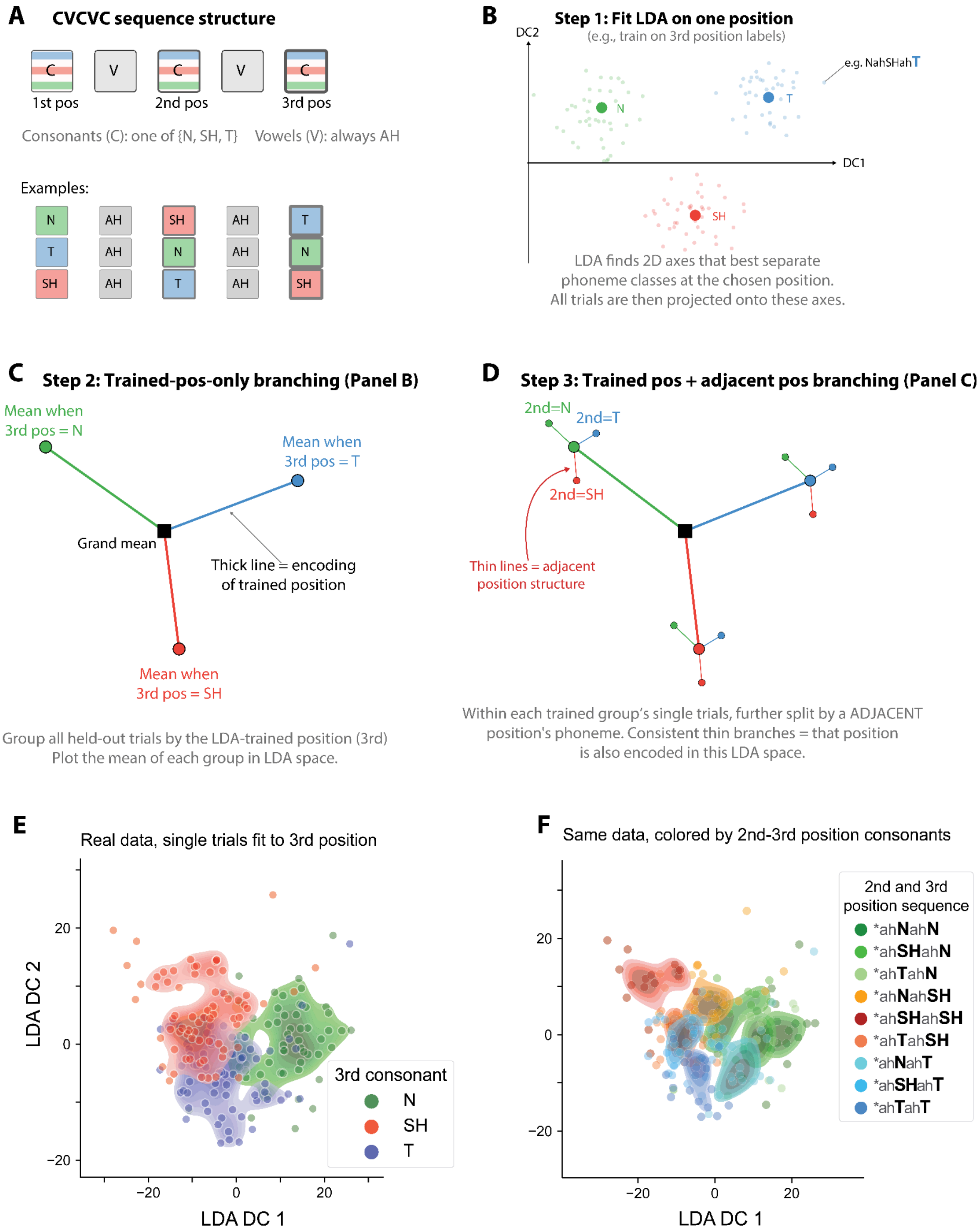

Figure S3: Explanation of the displacement vector analysis in LDA discriminant space (related to Figure 2BC). A) Schematic illustrating how full-factorial CVCVC sequences are operationalized as three consonant positions drawn from a three-consonant inventory. Vowels were fixed across all conditions. **B)** An LDA model is fit to decode a single sequence position

bioRxiv preprint doi: https://doi.org/10.64898/2026.04.27.721195; this version posted April 30, 2026. The copyright holder for this preprint (which was not certified by peer review) is the author/funder, who has granted bioRxiv a license to display the preprint in perpetuity. It is made available under aCC-BY 4.0 International license.

(here, the 3rd) from preparatory neural activity, yielding two discriminant components (DCs) that maximally separate the three consonant classes. Simulated held-out trials projected into this DC space are shown for illustration: individual trials (small markers) cluster by 3rd-position consonant identity and class means (large markers) are well separated. Trials span many conditions (e.g., NahSHahT) but are colored solely by 3rd-position consonant (e.g., /T/). **C)** Held-out trials are grouped and averaged by 3rd-position consonant identity. A displacement vector is computed for each class as the vector from the grand mean of all held-out trials to each class mean (thick lines), representing the direction in DC space associated with each 3rd-position consonant. **D)** To visualize the encoding of adjacent positions, displacement vectors for the 2nd position are computed. Trials are now grouped and averaged by both 2nd- and 3rd-position consonant identity, yielding nine group means. For each 3rd-position class mean (derived in **C**), a 2nd-position displacement vector is computed as the vector pointing from that 3rd-position mean to the corresponding 2nd–3rd joint mean (thin lines), isolating the contribution of 2nd-position identity independent of 3rd-position context. Consistent orientation of 2nd-position displacement vectors for the same consonant across 3rd-position contexts (e.g., all thin red lines pointing in a similar direction for 2nd-position /SH/) and alignment with the corresponding 3rd-position displacement vector (thick red line) indicates a shared neural representation of the same consonant across positions. Note: panels **B–D** depict simulated data for illustrative purposes only; corresponding real data are shown in panels **E–F** and Figure 2B–C. **E)** Real held-out single trials from T12 area i6v underlying Figure 2B–C, projected into the LDA DC space and colored by 3rd-position consonant identity. Each cluster appears multimodal rather than unimodal. Threshold crossing counts were summed over the 0.7s preparatory window preceding the go cue, following audio cue offset. **F)** Same data as **E**, now colored by joint 2nd- and 3rd-position consonant identity. The multimodality visible in **E** is partially explained by sub-clustering according to 2nd-position consonant identity, consistent with the shared representational structure described in Figure 2.

bioRxiv preprint doi: https://doi.org/10.64898/2026.04.27.721195; this version posted April 30, 2026. The copyright holder for this preprint (which was not certified by peer review) is the author/funder, who has granted bioRxiv a license to display the preprint in perpetuity. It is made available under aCC-BY 4.0 International license.

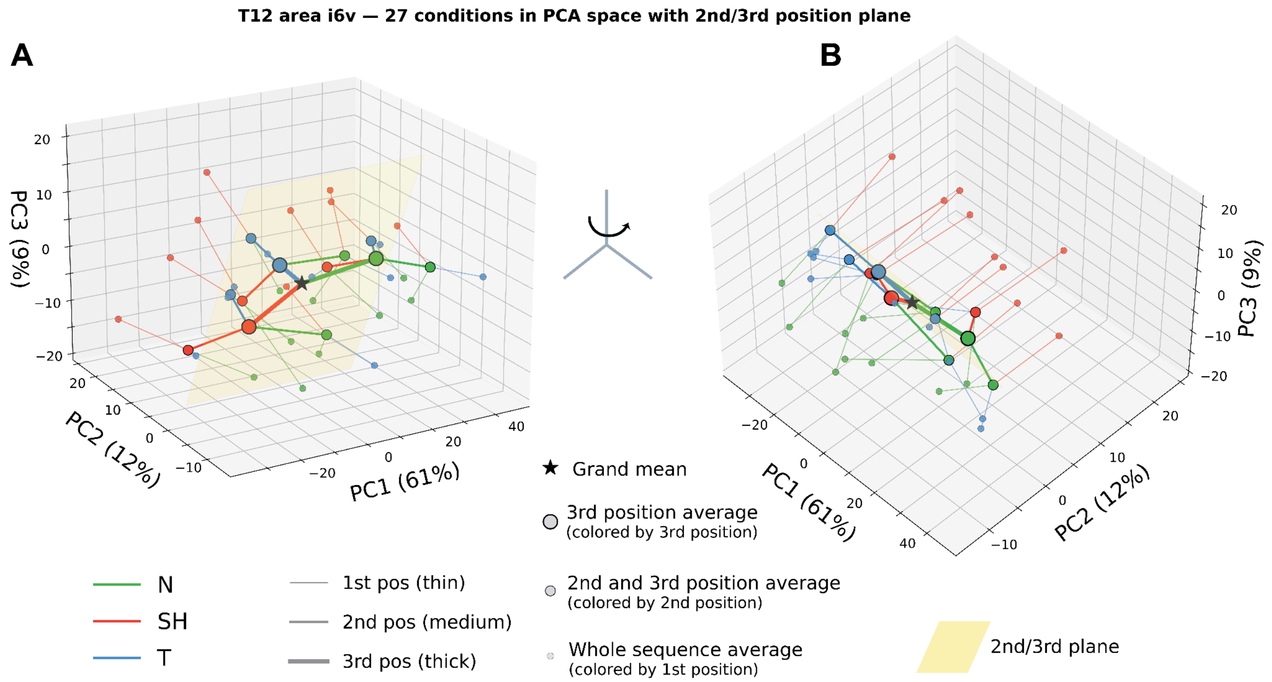

Figure S4: PCA-derived geometry of CVCVC sequences. A) Principal component analysis (PCA) provides an unsupervised approach to dimensionality reduction (compared to LDA), which is guaranteed to preserve the geometry of the original high-dimensional neural activity. PCA was performed on average neural activity (threshold-crossing counts over a 0.7s preparatory window from area i6v in T12) of whole sequences such that principal components are optimized to explain variance between average preparatory representations of phoneme sequences. Consonants subselected from {N, SH, T} to match Figure 2 resulting in 3^3=27 total conditions. The top three principal components (PCs) captured 81% of condition-mean variance and were retained for visualization. Displacement vectors for the 1st, 2nd, 3rd position are shown. See Fig. S3 for visual explanation of analysis, extended here to the 1st position. A similar structure arises, with a 2D plane capturing more than 90% of the variance of 2nd-3rd position sequences (yellow, similar to Figure 2BC). Within this plane, displacement vectors are consistently aligned for the 2nd and 3rd position (medium and thick lines). **B)** The same data as in **A** are shown from a rotated view orthogonal to the 2nd-3rd position plane. 1st phoneme representations are organized largely orthogonally to the 2nd-3rd position plane. Related to Figure 2.

bioRxiv preprint doi: https://doi.org/10.64898/2026.04.27.721195; this version posted April 30, 2026. The copyright holder for this preprint (which was not certified by peer review) is the author/funder, who has granted bioRxiv a license to display the preprint in perpetuity. It is made available under aCC-BY 4.0 International license.

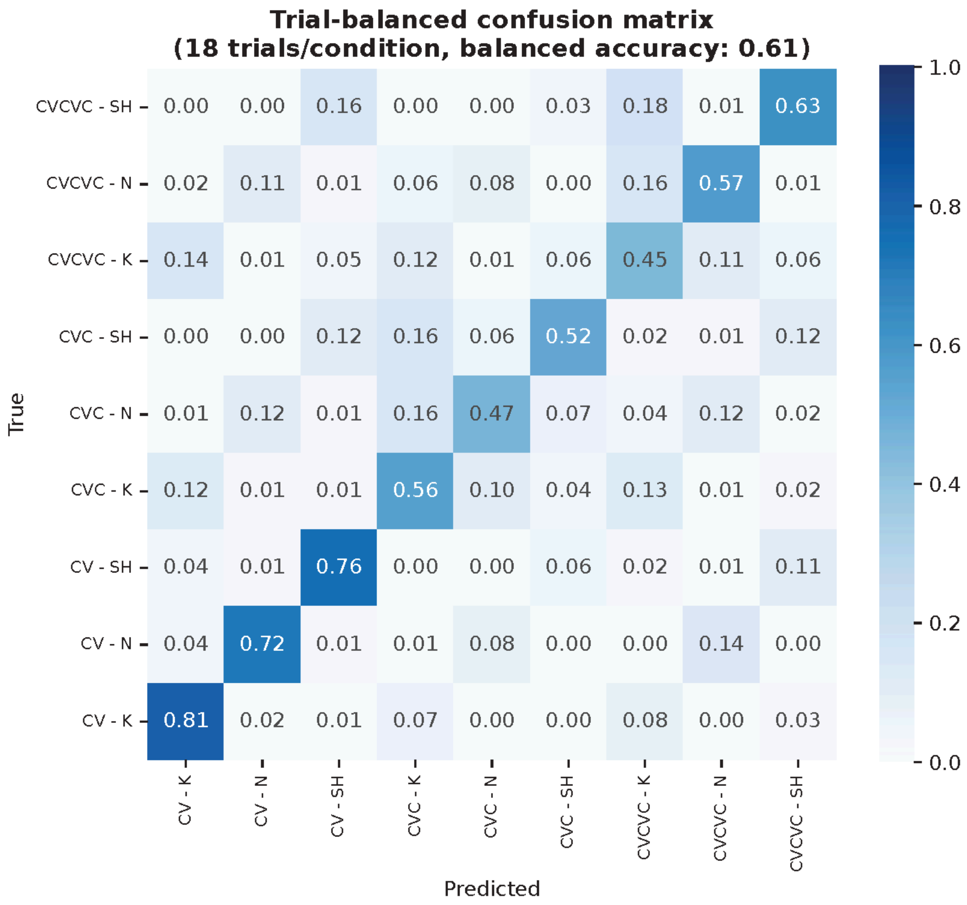

Figure S5: Trial-balanced decoding of sequence length and first-position phoneme identity from preparatory activity in T12 area i6v. In the factorial design used here, fixing trial counts per sequence condition results in fewer total trials for shorter sequence types, which contain fewer unique conditions (e.g., 3 CV conditions versus 27 CVCVC conditions for a 3-consonant inventory). The decoder in Figure 3B was therefore trained on all available trials, introducing class imbalance that may inflate apparent confusion among minority-class sequence lengths due to a difference in class probability. To assess whether the confusion pattern in Figure 3B reflects the underlying representational geometry rather than class imbalance, a trial-balanced decoder was fit after subsampling majority classes to match the trial count of the minority class. Decoding performance remained strong and broadly uniform across all sequence length × first-position phoneme classes, demonstrating that the confusion observed for shorter sequences in Figure 3B is due to trial imbalance.

bioRxiv preprint doi: https://doi.org/10.64898/2026.04.27.721195; this version posted April 30, 2026. The copyright holder for this preprint (which was not certified by peer review) is the author/funder, who has granted bioRxiv a license to display the preprint in perpetuity. It is made available under aCC-BY 4.0 International license.

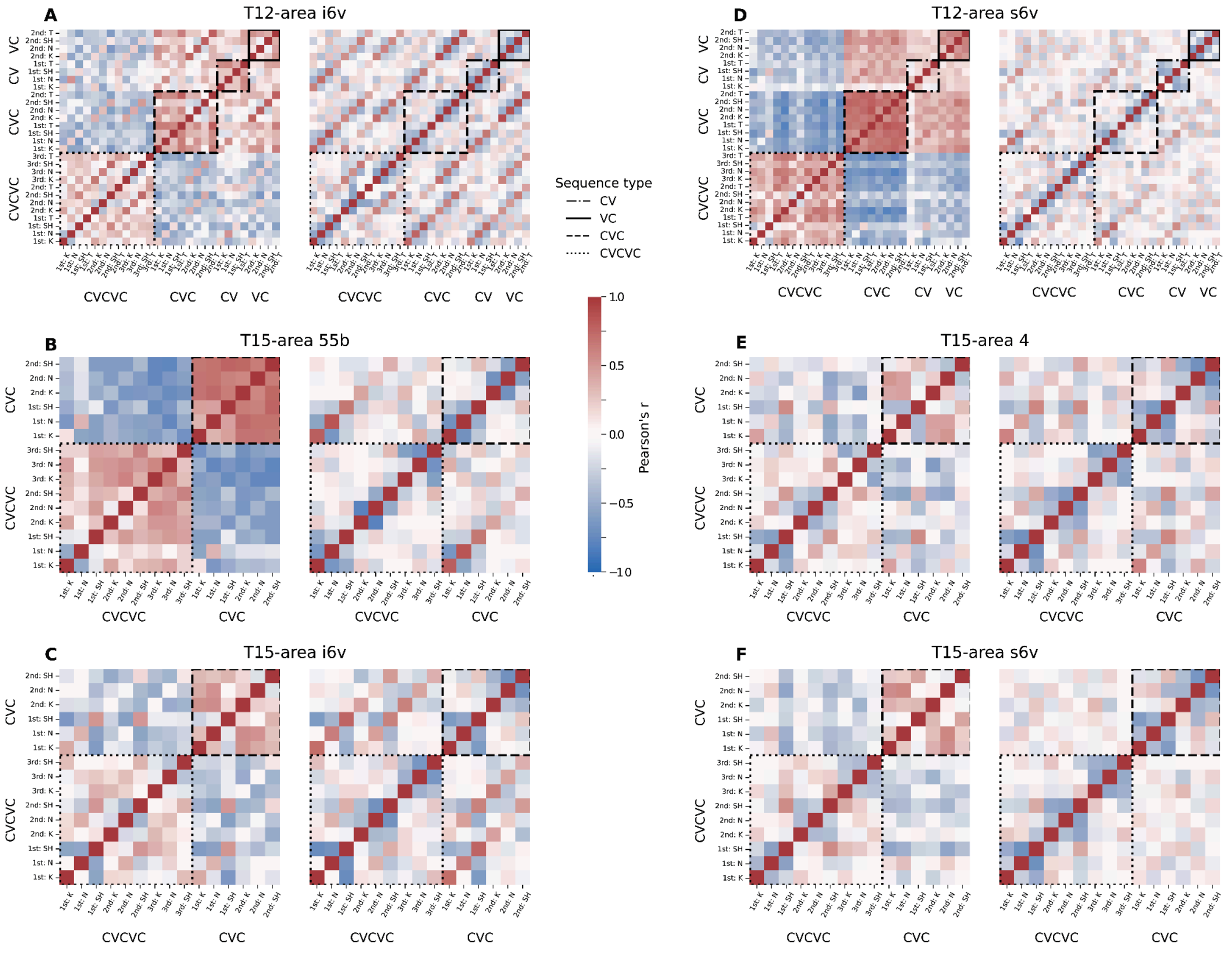

Figure S6: Like-sequence types are broadly correlated across phonemes and marginalizing out sequence-type reveals a shared phonetic code. Pairwise Pearson correlations between population activity vectors averaged over trials sharing the same sequence type, and phoneme identity in a specific sequence position. Each matrix entry reflects the correlation between two such averages — for example, the mean preparatory population state for the 2nd-position /K/ in CVC sequences versus the 1st-position /SH/ in CVCVC sequences — computed across spike-band power and threshold-crossing counts on all electrodes in a single microelectrode array during the preparatory window (0.6s prior to go cue). Diagonal squares outlined rectangles indicate entries sharing the same sequence type. Each pair of matrices show results for a single microelectrode array; Left: Raw correlation matrix. Right: Correlation matrix after subtracting the sequence-type mean population vector from each trial prior to averaging, isolating phoneme- and position-specific representational geometry from sequence-type-level offsets. Related to Figure 3.

bioRxiv preprint doi: https://doi.org/10.64898/2026.04.27.721195; this version posted April 30, 2026. The copyright holder for this preprint (which was not certified by peer review) is the author/funder, who has granted bioRxiv a license to display the preprint in perpetuity. It is made available under aCC-BY 4.0 International license.

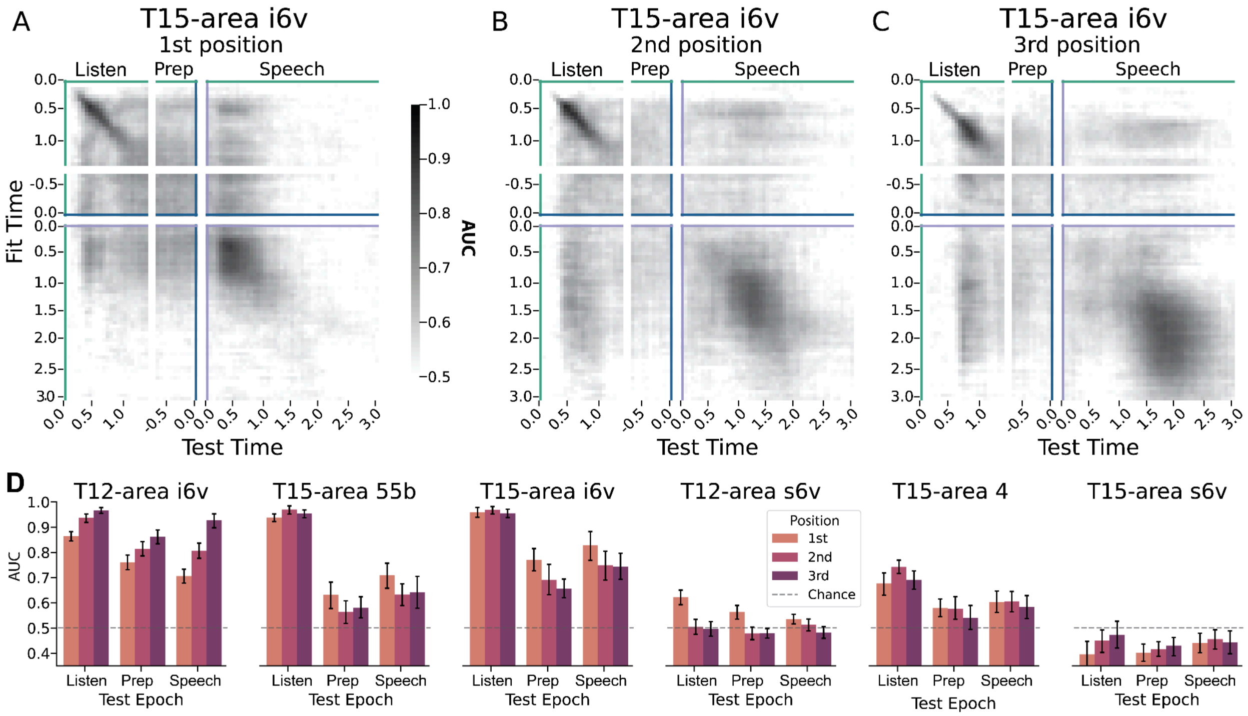

Figure S7: Cross-time generalization for T15 and per-position epoch generalization. A) Cross-time decoder generalization analysis of Figure 4D reproduced for T15-area i6v (1st position decoding performance). Decoders generalize broadly from listening to preparation and speech. Results were similar for 2nd (**B**) and 3rd (**C**) position decoders. **D)** Same cross-epoch decoding results as Figure 4G: decoders fit to predict between pairs of conditions from neural activity averaged over a 0.5s window of audio-cue listening and tested during all trial-epochs. Results here are plotted by sequence position (results in Figure 4G are for average over position). Error bars are 95% confidence intervals over decoders.

bioRxiv preprint doi: https://doi.org/10.64898/2026.04.27.721195; this version posted April 30, 2026. The copyright holder for this preprint (which was not certified by peer review) is the author/funder, who has granted bioRxiv a license to display the preprint in perpetuity. It is made available under aCC-BY 4.0 International license.

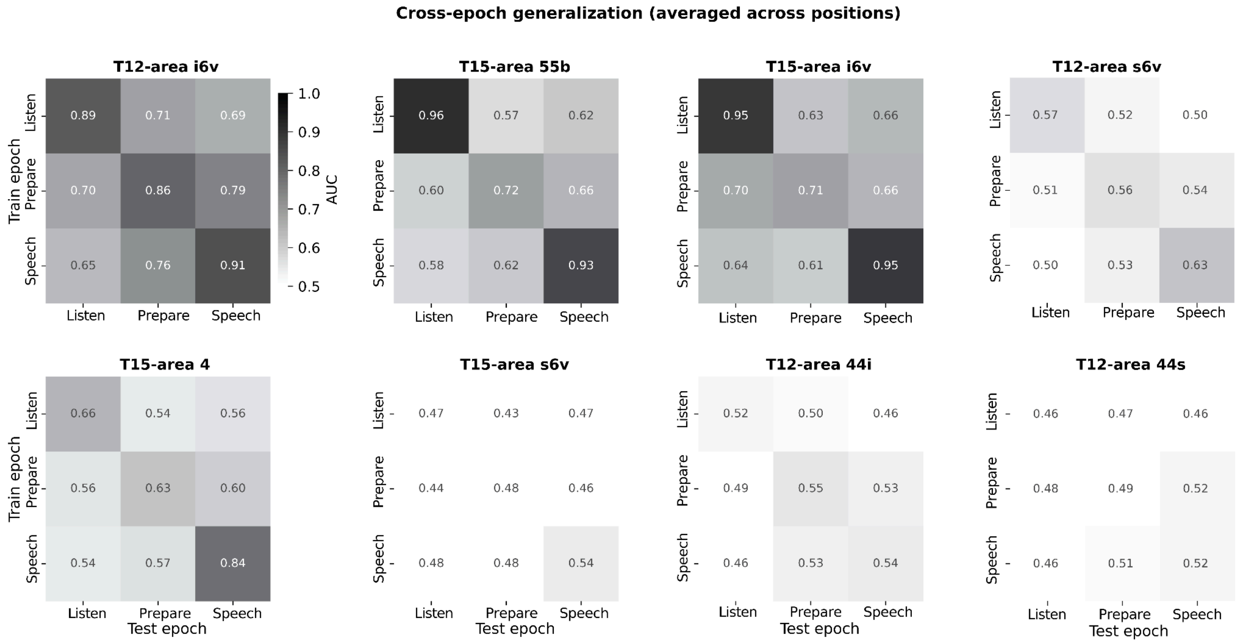

Figure S8: Cross-epoch generalization of phoneme decoding across all trial-epoch pairs, per array. Extension of Figure 4E in which decoders are trained and tested on all combinations of task epochs (Listen, Prepare, Speech), rather than training on Listen only. Each panel shows decoding performance (AUC, chance = 0.5) for a single array; rows indicate training epoch, columns indicate test epoch. Diagonal entries reflect within-epoch performance; off-diagonal entries reflect cross-epoch generalization. Values are averaged across sequence positions.
